# Research on the Influence of Temperature on the Stress–Electromagnetic Characterization of Radiation-Resistant Robotic Drive Steel Cables

**DOI:** 10.3390/ma18204686

**Published:** 2025-10-13

**Authors:** Tong Wu, Linlong Ding, Yingchun Chen, Jie Yang, Renjie Nie, Fengjuan Chen, Chuan Zhang, Jiahao Wu

**Affiliations:** 1 CNNC Hexin Information Technology (Beijing) Co., Ltd., Beijing 100091, China; 2 The Faculty Nuclear Industry X Intelligence Laboratory, Beijing University of Technology, Beijing 100124, China; 3 College of Mechanical and Energy Engineering, Beijing University of Technology, Beijing 100124, China

**Keywords:** magnetoelastic effect, sensor, steel cable stress, temperature, backpropagation neural network model

## Abstract

During the operation of steel cable-driven radiation-resistant robots in nuclear industrial environments, the tensile force of a steel cable is influenced by temperature variations, which can cause significant detection errors. To address this problem, this study proposes a temperature-compensated axial force characterization method for steel cables based on the magnetoelastic effect, aiming to ensure the measurement accuracy of magnetoelastic sensors. The principle of the magnetoelastic measurement method involves magnetizing the steel cable. When subjected to tensile forces, the magnetization characteristics of the steel cable change, thereby altering the detection signal of the magnetoelastic sensor. By analyzing the relationship between steel cable tension and variations in the detection signal, effective force measurement can be achieved. First, experiments are conducted to investigate the influence of temperature on the detection signals of a magnetoelastic sensor under zero-load conditions. Then, additional tests are performed to examine the combined effects of a tensile force and temperature on the sensor’s signals. Finally, based on the experimental data, axial force prediction models are constructed using both surface fitting and a backpropagation neural network (BPNN). The results demonstrate that, compared to the resistance values, inductance exhibits superior stability under temperature variations. In the temperature range of 20–50 °C, the inductance variation is approximately 0.15 μH, which indicates improved suitability for characterizing the axial force of steel cables. It is also shown that under isothermal conditions, the inductance increases linearly with the applied tensile force, exhibiting a slope of approximately 0.025 μH/kN. Both the surface fitting-based and BPNN-based axial force prediction models demonstrate high accuracy, with absolute prediction errors consistently below 5% compared to actual data.

## 1. Introduction

**Figure 1:**
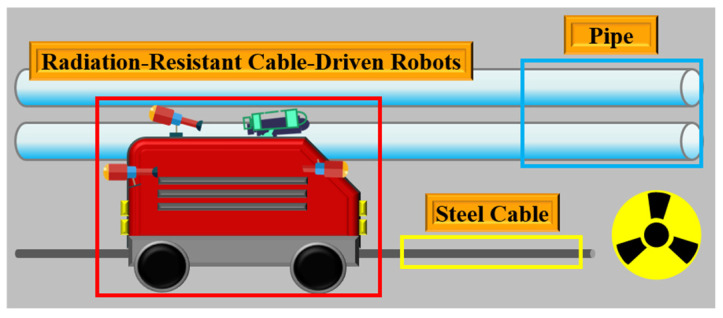
Schematic diagram of the steel cable-driven radiation-resistant robot.

Steel cable-driven radiation-resistant robots have been commonly used as inspection equipment in the nuclear industry. They are mainly deployed in high-radiation, high-temperature, and high-humidity environments, such as reactor chambers and spent fuel pools, to replace manual operations for hazardous tasks, including inspection, maintenance, and sampling. A schematic diagram of a steel cable-driven radiation-resistant robot is presented in Figure 1. As the driving medium of radiation-resistant robots, steel cables experience material degradation and mechanical damage when exposed to the elevated temperature conditions of nuclear industrial environments during prolonged operation. This damage could cause mission interruptions or even compromise nuclear safety systems. The stress distribution state of a steel cable is closely related to the safety performance of equipment, making stress detection essential [1]. Under the influence of long-term static and dynamic alternating loads, combined with environmental factors (e.g., temperature fluctuations in nuclear facilities) and human influences (e.g., improper maintenance), a steel cable is susceptible to wear, corrosion, and fatigue damage [2,3]. This highlights the importance of ensuring robot operation safety. Such deterioration can trigger a failure of structural components, which may result in catastrophic infrastructure malfunction. In addition, unpredictable mechanical failures can cause accidents, casualties, and significant economic losses [4,5]. To prevent functional disruptions and potential structural failures, structural health monitoring (SHM), as an emerging concept in engineering, aims to identify damage and degradation by continuously monitoring structural behavior and assessing performance under operational loads [6].

Currently, the mainstream methods for measuring steel cable tension include hydraulic pressure gauge measurement [7], pressure sensor-based detection [8], electrical resistance strain gauge technique [9], fiber Bragg grating sensing [10], vibration frequency analysis [11], computer vision-based measurement [12], and the magnetoelastic effect method [13]. Among them, the magnetoelastic effect method has been the most widely used technique for stress detection in structural steel cables and industrial equipment due to its non-contact nature, long service life, and suitability for long-term monitoring [14].

Grimes et al. provided a detailed exposition on the magnetoelastic effect. This effect describes the coupling relationship between the mechanical state and the magnetic state in ferromagnetic materials (such as the steel used in bridge cables): changes in the internal magnetization state of the material can induce elastic deformation of its physical dimensions, which is referred to as the direct magnetoelastic effect; conversely, the application of external mechanical stress (such as tension or compression) can alter the material’s magnetization intensity, permeability, and other parameters, which is termed the inverse magnetoelastic effect [15]. The fundamental principle of the magnetoelastic-based steel cable tension detection process is that when a steel cable is subjected to tensile stress, its magnetic properties vary with the applied stress due to the ferromagnetic nature of the cable material [16]. This effect becomes particularly pronounced when a steel cable is magnetized. By measuring the magnetization curve under tensile loading and extracting the slope, which is defined as a ratio of magnetic field intensity to magnetic flux density, the relative permeability of the steel cable material can be determined [17]. The core task of the magnetoelastic effect method is to determine the relationship between relative permeability and the tensile stress of steel cables, enabling the quantification of cable tension [18].

Magnetoelastic tension sensors offer distinct advantages over conventional strain gauges, including significantly higher sensitivity to stress variations—particularly in high-stress environments—and the ability to directly measure absolute stress rather than strain. These sensors function passively and wirelessly, eliminating the need for onboard power and enabling non-contact remote monitoring, which makes them highly suitable for applications in harsh or inaccessible environments. Despite these benefits, they are hampered by notable limitations such as a strong temperature dependence that leads to measurement drift, hysteresis under dynamic loading conditions, and vulnerability to external magnetic interference. However, the most significant limitation of magnetoelastic sensors is their strong susceptibility to temperature variations, which substantially affects measurement accuracy. Generally, the magnetic signals of ferromagnetic materials exhibit complex temperature-dependent behaviors influenced by multiple factors, including material composition, applied magnetic field intensity, and temperature gradient direction. Therefore, to investigate the underlying mechanisms of temperature effects on ferromagnetic materials, researchers have conducted numerous theoretical studies [19,20], simulation analyses [21,22], and experimental validations [23,24,25].

Wang et al. [26] introduced a generalized coupled magneto-thermo-elastic free energy model for Terfenol-D rods, which incorporates Weiss molecular field effects based on thermodynamic theory. This model also adopts Jiles–Atherton’s magneto-mechanical coupling hypothesis. Building upon previous work, Xiao et al. [27] introduced eddy current loss theory and proposed a one-dimensional nonlinear thermo-magneto-elastic hysteretic constitutive model for giant magnetostrictive materials. Considering the influence of temperature on magnetoelastic measurements, Zhou Jianting et al. [28] proposed a temperature compensation method based on the genetic algorithm backpropagation neural networks for magnetoelastic sensors. The temperature compensation method reduced the relative error of stress monitoring to 3.3%. These findings indicated the need for systematic research on the effects of temperature. Research in this field should focus on the stress characterization of radiation-resistant robotic drive steel cables.

To address the current challenges in the field, this study designs a detachable sleeve-type sensor based on the magnetoelastic effect principle and constructs an experimental platform to characterize the axial tension of steel cables under the influence of temperature. In addition, repeatability tests are conducted to verify the feasibility of the tension characterization approach. Moreover, experimental data are used to develop two predictive models: a surface fitting-based axial load prediction model and a backpropagation neural network-based model for axial load forecasting (BPNN). Both models are validated through performance testing. The prediction errors of the models, when compared to actual force values, fall within ±5%, demonstrating the strong compensation capability of the proposed method. The findings of this study could contribute to a more comprehensive understanding of the effects of temperature on the electromagnetic characterization of tensile stress of steel cables, particularly for radiation-resistant robotic applications. In addition, the proposed sensor design and predictive modeling method can provide valuable insights for improving the accuracy and reliability of steel cable tension monitoring in high-temperature nuclear environments. This study proposes a temperature-compensated axial force characterization method for steel cables based on the magnetoelastic effect, presenting a novel approach for detecting driving steel cables in radiation-resistant robots in nuclear industrial environments. The research contributes to the theoretical foundation for inspecting drive steel cables in radiation-resistant robotic systems in nuclear industrial environments.

## 2. Experimental Results

### 2.1. Materials

**Figure 2:**
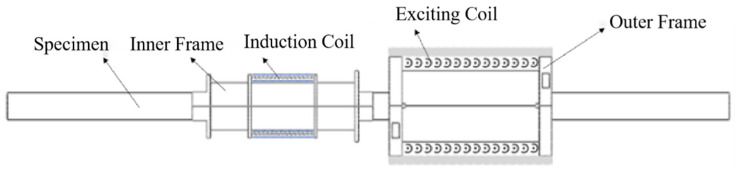
The schematic diagram of a sleeve-type sensor structure.

This study simulated the influence of temperature on electromagnetic characteristics of drive cables used in radiation-resistant robots within nuclear industrial environments. The thermal management system, comprising the custom-fabricated foamed silicone rubber tubing and flexible heating tube, was specifically designed and sized to match the precise dimensions of the test specimen and experimental setup. Temperature control was achieved by encapsulating a steel cable with a foamed silicone rubber hose and a flexible heating tube. The foamed silicone rubber hose provided thermal insulation, whereas the flexible heating tube enabled temperature regulation. The experimental materials are shown in Figure 2, and their parameters are listed in Table 1. The flexible heating tube consists of an internal heating wire, a glass fiber blanket, and an insulating layer. In the experiment, a single steel strand was used. Each strand is composed of seven steel wires twisted together, without any surface coating. The detailed parameters of the steel strand are provided in Table 2. The steel strand specimen used in the study was a single-diameter (15.2 mm) steel strand, procured from a specialized manufacturer (PRC) and compliant with the Chinese national standard GB/T 30826-2014 [29] (“Technical Conditions for Steel Strand Cables in Cable-Stayed Bridges”).

### 2.2. Test Methods

In the experiments, a detachable sleeve-type sensor was used to characterize the axial tension of a steel cable. The selected sensor’s structure primarily consisted of an outer excitation coil and an inner induction coil, which were integrated with a custom-built magnetoelastic instrument and an inductance-capacitance-resistance (LCR) meter to construct a complete excitation-measurement system.

The magnetoelastic instrument was connected to the excitation coil to generate pulsed signals, which were then used to magnetize the steel cable enclosed within the sensor assembly. Simultaneously, the LCR meter, which was linked to the induction coil, applied alternating current (AC) signals to measure the coil’s electrical responses (i.e., the inductance and resistance values). A schematic diagram of the sleeve-type sensor’s structure is displayed in Figure 2.

**Figure 3:**
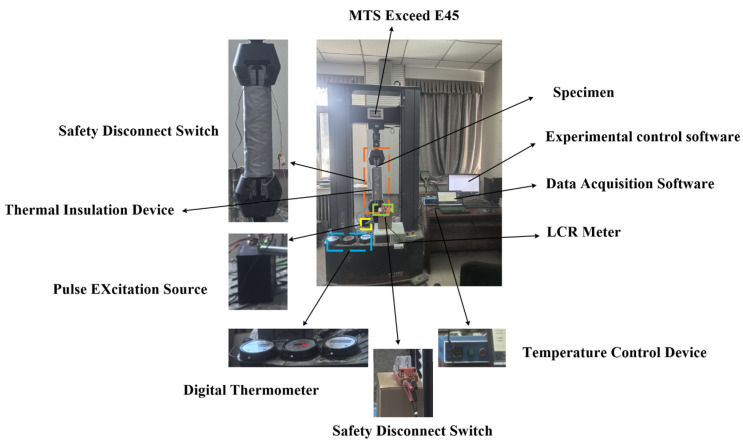
Photo of the experimental platform for axial tension characterization of steel cables under temperature influence.

The axial tensile loading of the steel cable was conducted using an MTS Exceed E45 electromechanical universal testing machine, manufactured by MTS Systems Corporation, Eden Prairie, MN, USA, and distributed to China by MTS Industrial Systems (Shanghai, China) Co., Ltd., 37. This system was integrated with a sleeve-type sensor, an excitation-measurement unit, and a temperature control device, establishing a comprehensive test platform. The test platform was designed to evaluate axial tension performance, and the testing process was conducted under temperature-compensated conditions. The detailed experimental configuration is depicted in Figure 3.

The electronic universal testing machine employed was the MTS Exceed 45, manufactured by MTS Industrial Systems (Eden Prairie, MN, USA) Co., Ltd., which offers high reliability and ease of operation. The testing system incorporates a high-speed, low-vibration motor drive and an integrated digital closed-loop control system, capable of performing tests under force, displacement, or strain control within a range of 5 N to 300 kN. The control software, TestSuite TW (Eden Prairie, MN, USA), meets requirements for specialized or complex testing protocols, is user-friendly, and satisfies the demands of rapid and efficient quality assurance and quality control testing. The data acquisition software was a self-developed upper-computer program designed to interface with an LCR meter. This software continuously sends commands via a serial port to modify the frequency and receive data, with the frequency increasing logarithmically. After each frequency change, multiple sets of inductance and resistance values are read, averaged, and stored. The acquired data can be plotted graphically in real time, and the average inductance and resistance values across different frequencies are ultimately exported to an Excel spreadsheet.

The experimental parameters used for the temperature influence analysis on the detection signal under zero-tension conditions are listed in Table 3.

**Figure 4:**
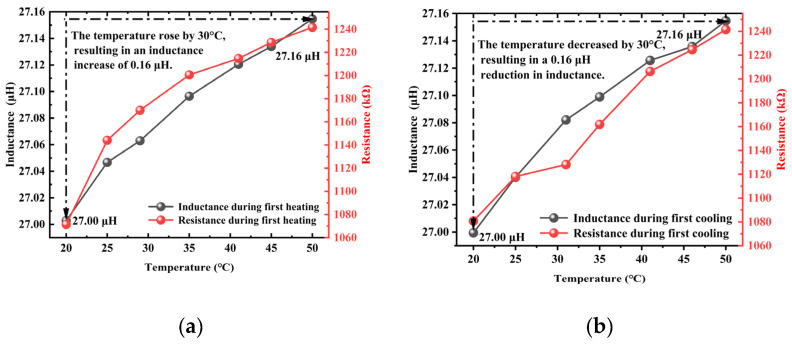
The inductance and resistance curves obtained during the heating and cooling processes in the first trial: (**a**) heating, (**b**) cooling.

**Figure 5:**
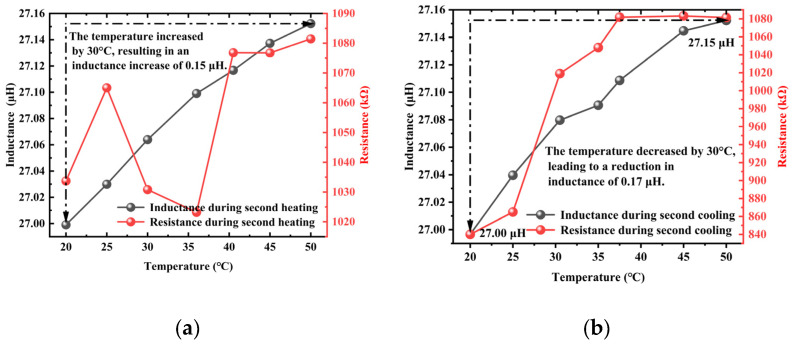
The inductance and resistance curves obtained during the heating and cooling processes in the second trial: (**a**) heating, (**b**) cooling.

**Figure 6:**
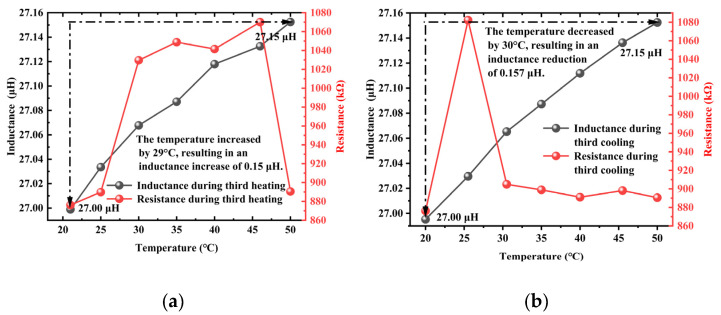
The inductance and resistance curves obtained during the heating and cooling processes in the third trial: (**a**) heating, (**b**) cooling.

The curves of inductance and resistance values obtained in three separate trials during the heating and cooling processes are presented in Figure 4, Figure 5 and Figure 6. In the experiments, the resistance value exhibited significant and anomalous abrupt changes, showing inconsistent trends across the three trials. This indicated that under the combined influence of temperature and other affecting factors, the measured resistance value was subject to considerable errors, making it unsuitable for subsequent characterization of the axial tension in steel cables. In contrast, the inductance value demonstrated favorable linearity and repeatability throughout the three trials. Therefore, the inductance value was selected as a characteristic parameter for characterizing the axial tension in steel cables in subsequent axial tension tests.

To investigate the influence of temperature on detection signals under tensile conditions, this study conducted experiments to measure the inductance of steel cables at different temperatures and tensile loads. In the experiments, both temperature and tensile load were regarded as independent variables, with value ranges of 20–50 °C and 20 kN–100 kN, respectively. To ensure the reliability of the experimental results, three repeated tests were conducted under identical conditions. The test parameters used to examine the effects of temperature on detection signals under tensile loading are summarized in Table 2.

## 3. Results and Discussion

### 3.1. Analysis of Temperature Effects on Inductance Under Tensile Load Absence

**Figure 7:**
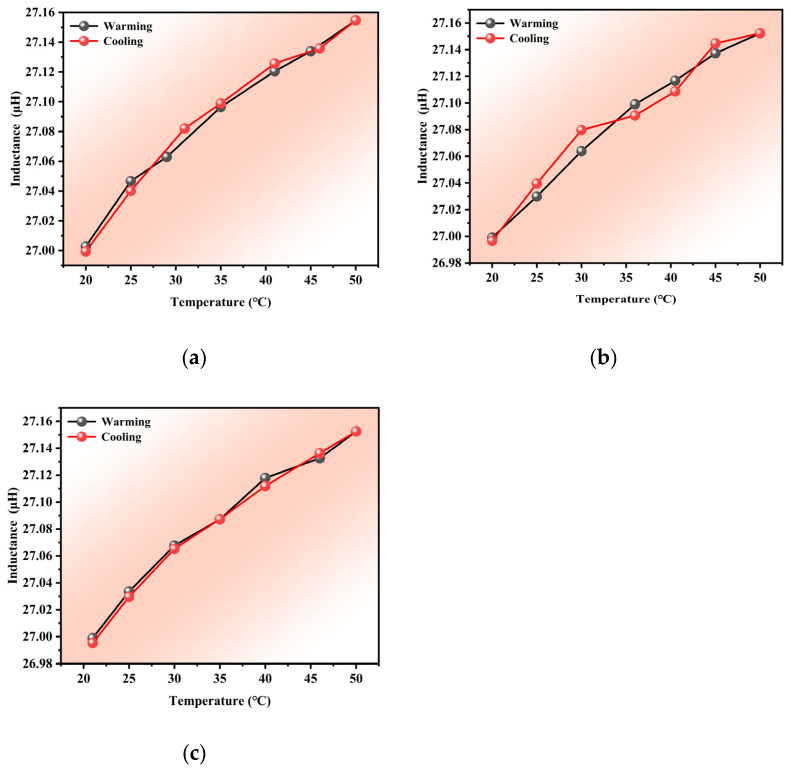
The inductance curves obtained in the three temperature-variation tests: (**a**) the first test, (**b**) the second test, (**c**) the third test.

The results of three heating and cooling tests are presented in Figure 7, where it can be observed that the inductance of the unstressed steel cable increased gradually with temperature, showing a relatively small overall variation amplitude of approximately 0.15 μH. The cooling trend essentially mirrored the heating trend, demonstrating good repeatability and reversibility without significant hysteresis. Compared to the temperature-dependent resistance curve shown in Figure 5, the inductance curve presented in Figure 7 was less affected by temperature under the same environmental conditions, showing a much smaller variation amplitude than the resistance curve while maintaining excellent reversibility.

Under thermal loading, the electrical resistance of the steel cable exhibits more pronounced and abrupt variations compared to the relatively stable inductance, which can be attributed to distinct underlying physical mechanisms. The resistance change is primarily influenced by four factors. First, the positive temperature coefficient of the metallic material’s resistivity, where increased lattice vibration amplitudes at elevated temperatures enhance electron scattering, leading to a near-linear rise in resistivity. Second, interfacial contact resistance changes caused by differential thermal expansion between materials such as the steel strand and sensor electrodes, resulting in microscopic gaps or contact pressure variations that induce abrupt resistance jumps. Then, surface oxidation effects accelerated by higher temperatures, particularly in copper or steel elements, where even thin oxide layers introduce additional and often nonlinear interfacial resistance. Finally, non-uniform thermal expansion altering the geometric dimensions of conductive paths, with localized stress concentrations or anisotropic behavior occasionally triggering sharper resistance changes. In contrast, inductance remains comparatively stable due to its dependence on bulk magnetic properties and the magnetoelastic effect, which responds to the average stress and magnetization state within the material. These magnetic characteristics evolve more gradually with temperature, making inductance less sensitive to acute interfacial and surface phenomena that dominate resistance behavior.

**Figure 8:**
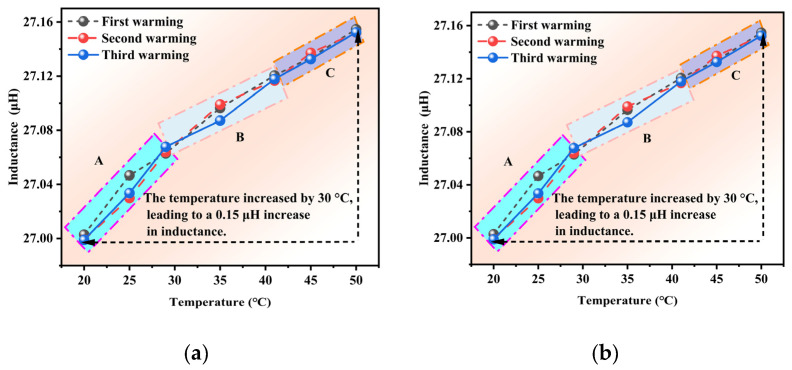
Inductance variations as a function of temperature. (**a**) heating, (**b**) cooling.

The influence of temperature on inductance is presented in Figure 8. As shown in Figure 8a, the inductance value increased with the temperature across all experimental trials and could be categorized into three distinct regions. Based on the difference in inductance values between the three experiments, the variation with temperature can be divided into three regions: A, B, and C. The differences in inductance value between the three tests were as follows: Region A (0.017 μH) > Region B (0.012 μH) > Region C (0.005 μH). A 30 °C temperature increase produced a 0.15 μH. The experimental results revealed distinct temperature-dependent inductance characteristics across the three defined regions. Region A exhibited the most pronounced inductance variations, which could correspond to the temperature-sensitive zone of either the magnetic core’s permeability or the conductor’s resistivity. In contrast, Region C demonstrated significantly attenuated variations, indicating stabilization of both the material properties and structural deformation in this region. In addition, increased conductor resistivity, particularly in copper windings, enhanced the skin effect, reducing effective conduction area and consequently increasing the equivalent inductance. Furthermore, thermal expansion induced micro-deformations in the coil formers and windings, thus modifying inter-turn capacitance and magnetic circuit air gaps, which jointly affected inductance parameters. The non-uniform temperature distributions generated localized stress variations that exacerbated inductance dispersion.

The coil former is made of nylon (thermal expansion coefficient ≈ 80–100 × 10^−^^6^/°C), while the copper winding has a coefficient of approximately 17 × 10^−^^6^/°C. The significant mismatch in their thermal expansion behaviors induces interfacial stresses during temperature changes, causing the former to expand more than the winding. This differential expansion alters the inter-turn spacing and distorts the magnetic circuit air gap, leading to measurable changes in inductance.

In addition, the core material has a relatively low thermal expansion coefficient (≈10 × 10^−^^6^/°C). When combined with the much higher expansion of the nylon former, non-uniform temperature distribution produces localized stress gradients, particularly near material interfaces. These stresses not only contribute to geometric changes (e.g., increased magnetic reluctance due to widening air gaps) but may also leave residual strains after thermal cycling, further dispersing inductance readings over repeated experiments.

As illustrated in Figure 8b, the inductance value demonstrated a consistent decreasing trend with temperature reduction across all experimental trials, exhibiting three distinct characteristic regions. The inductance variations in the three tests followed a specific pattern of first decreasing, then increasing, and finally decreasing again. Notably, at 40 °C, the observed inductance variation reached 0.017 μH. The experimental data further revealed that a 30 °C temperature decrease corresponded to an inductance reduction of 0.16 μH.

The results showed that lower temperatures reduced the resistivity of copper windings, diminishing the skin effect. This yielded a more uniform current distribution and increased the effective conduction cross-sectional area, ultimately decreasing the equivalent inductance. Second, enhanced domain alignment in ferrite-based soft magnetic materials at reduced temperatures decreased the initial permeability, consequently reducing the inductance value. Third, thermal contraction of coil formers and windings induced microstructural modifications, including changes in inter-turn spacing and adjustments of magnetic circuit air gaps. All these structural alterations jointly influenced the inductance parameters. Furthermore, non-uniform material contraction might introduce mechanical stresses, causing nonlinear variations in the inductance value.

The observed inductance variation of 0.017 μH at 40 °C suggested that this temperature range might correspond to a critical transition point for either material phase change or structural response. During the overall 30 °C cooling process, the inductance decreased by 0.16 μH, demonstrating approximate symmetry with the heating cycle measurements. This near-symmetric behavior indicated that the temperature dependence of inductance exhibited partial reversibility, while the measurable difference confirmed the presence of thermal hysteresis effects. The observed hysteresis could originate from irreversible microstructural modifications or residual stresses accumulated during thermal cycling. This was consistent with the nonlinear variation patterns observed in different temperature regions.

The observed behavior suggested a non-monotonic relationship between temperature changes and inductance variations during the cooling cycles that could potentially have different underlying physical mechanisms compared to the heating process shown in Figure 8a. The measured 0.16 μH change represents a 6.7% variation relative to the baseline inductance value, which highlights the significant temperature dependence of the system’s electromagnetic properties.

### 3.2. Analysis of Temperature Effect on Inductance Under Tensile Load

**Figure 9:**
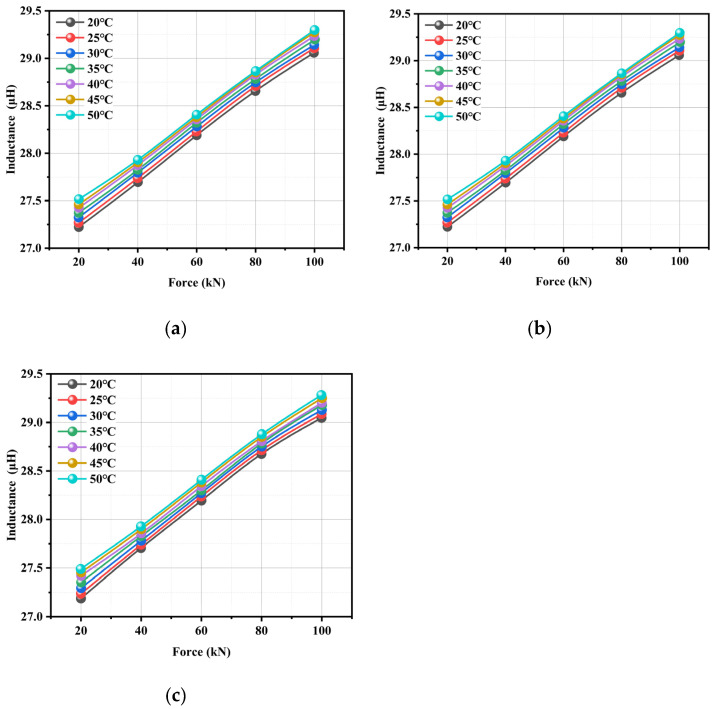
Inductance variations as a function of force: (**a**) the first test, (**b**) the second test, (**c**) the third test.

The inductance variations as a function of force are presented in Figure 9. The results demonstrated that the inductance value increased systematically from approximately 27.25 μH to 29.25 μH with an increasing tensile force under constant temperature conditions. Microstructurally, elastic lattice deformation under tensile stress modified the material’s magnetic permeability characteristics, but geometrically, the decrease in steel cable diameter and the elongation of the helical pitch during stretching significantly altered the electromagnetic parameters. The least-squares regression analysis yielded a linear fit with exceptional correlation (R^2^ > 0.99), which validated inductance as a precise indicator of tensile state. The measured force sensitivity of 0.025 μH/kN demonstrated robust performance across the specified thermal range, demonstrating the proposed method’s reliability for structural health monitoring applications.

The inductance exhibited a gradual increase with temperature, demonstrating a stable variation amplitude of (0.2–0.3) μH under a constant tensile load with a 30 °C temperature rise. This phenomenon indicated that when the steel cable inductance increased with temperature, its sensitivity to temperature variation (within the 10 °C range) remained significantly lower compared to the force dependence. Further, enhanced thermal motion in the magnetic field at elevated temperatures increased the material’s effective permeability. Finally, microscale geometric modifications occurred due to thermal expansion.

Under isothermal conditions, the steel cable’s inductance had a well-defined linear relationship with the applied tensile force, which was characterized by a systematic increase in inductance with increasing load. The experimental results revealed that limited temperature variations had a minimal influence on the inductance value; this was demonstrated by the essentially parallel nature of inductance-force curves across different temperature gradients. The observed parallel shift in inductance–force curves across temperature gradients—indicative of minimal temperature–load coupling—can be attributed to multiple underlying mechanisms. Firstly, the influence of mechanical load operates predominantly through the magnetoelastic effect, where stress alters magnetic permeability via domain rotation and deformation-induced anisotropy. In contrast, temperature impacts inductance mainly through thermal expansion and changes in intrinsic magnetic properties such as saturation magnetization. These mechanisms function largely independently, leading to additive rather than coupled effects. Secondly, within the studied range (20–50 °C), both the thermal expansion of materials and the stress-induced changes in magnetic permeability exhibit approximately linear behavior. The superposition of these linear responses results in a parallel displacement of the curves with temperature, without significant slope variation. Thirdly, the sensor structure is designed to limit nonlinear interactions at material interfaces; thermal expansion does not substantially disrupt stress distributions caused by axial loading. Finally, the operating temperatures remain well below the Curie point of the ferromagnetic material, ensuring stable and reversible magnetic responses without irreversible transitions. This decoupling facilitates straightforward temperature compensation in practical applications. This parallelism confirmed the absence of significant force–temperature coupling effects in the electromechanical response.

Physically, thermal variations predominantly alter the material’s intrinsic magnetic properties, while tensile forces concurrently modify both geometric and magnetic parameters. Multiple experiments have validated that the regular exhibited a certain degree of similarity, showing inter-experimental variations of less than 5% in sensitivity coefficients.

### 3.3. Analysis of Tensile Load Effect on Inductance at Different Temperatures

According to the Joule effect [30], the relationship between stress and magnetic permeability can be expressed as follows:

(1) $$\sigma =E\varepsilon =E\frac{\Delta l}{l}=E\frac{3\lambda_{s}M_{s}}{2K_{u}}\Delta M\sin^{2}\theta_{0}\cos\theta_{0}$$

where Δ*M* is the variation in magnetization intensity; *ε* is the axial deformation; *E* is the elastic modulus; *l* is the steel cable length; Δ*l* is the change in the steel cable length under tension; *λ*_s_ is the axial deformation constant; *M*_s_ is the maturation magnetization intensity; *K_u_* is the uniaxial magnetic anisotropy constant; *θ*_0_ is the angle between the magnetic field direction and the easy magnetization axis.

The variation in magnetization intensity (Δ*M*) is directly related to the product of the change in material permeability and the excitation current [28]. Substituting this relationship into Equation (1) yields:

(2) $$\sigma =E\frac{3\lambda_{s}M_{s}}{2K_{u}}\Delta (\mu -\mu_{0})\frac{nI}{l_{w}}\sin^{2}\theta_{0}\cos\theta_{0}$$

where *μ* is the magnetic permeability; *μ*_0_ is the permeability of free space; *n* is the number of coil turns; *I* is the excitation current; *L_w_* is the effective magnetic path length.

The relationship between magnetic permeability and inductance can be expressed by:

(3) $$L=\left(\frac{\mu A_{f}+\mu_{0}A_{air}}{l_{w}}\right)n^{2}$$

where *A_f_* is the cross-sectional area of the steel cable, and *A_air_* is the cross-sectional area of the air gap.

By integrating Equations (1)–(3), the relationship between the stress and inductance can be expressed by:

(4) $$F=E\frac{3\lambda_{s}M_{s}}{2K_{u}}I\left(\frac{L}{n}-\frac{n}{l_{w}}A\mu_{0}\right)\sin^{2}\theta_{0}\cos\theta_{0}$$

**Figure 10:**
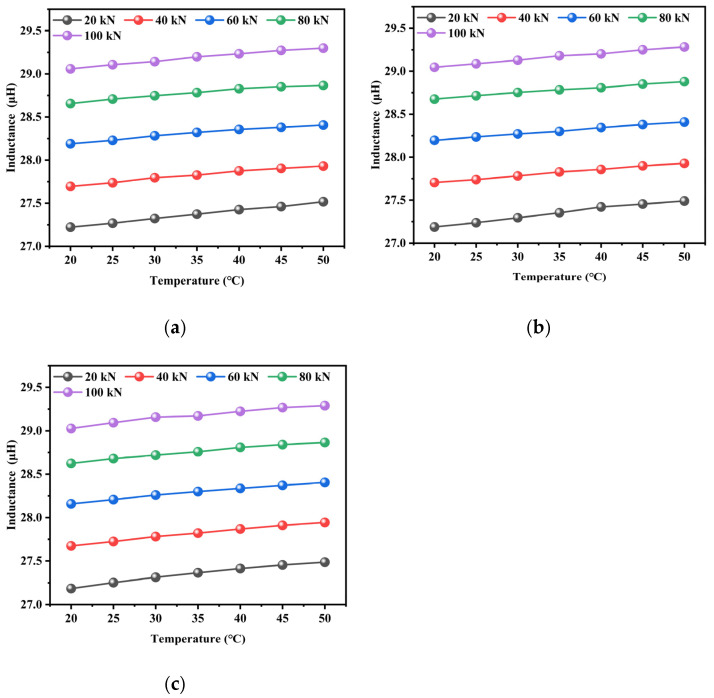
Influence of temperature on the inductance value under various tensile loads: (**a**) the first test, (**b**) the second test, (**c**) the third test.

The influence of temperature on the inductance value under different tensile loads is presented in Figure 10, where it can be seen that the inductance value showed a significant increasing trend with the temperature. Under a constant tensile load of 20 kN, a 250% temperature increase resulted in an inductance increment of 0.26 μH. Particularly, the incremental change in inductance between adjacent temperature intervals exhibited a progressive attenuation with continual temperature increase, and a consistent pattern was observed across various tensile load conditions. The enhanced domain wall mobility caused by thermal activation increased material permeability. Thermally induced geometric dimensional changes in the steel cable were observed, as well as high-temperature relaxation of internal residual stresses that modified domain alignment configurations. The observed reduction in the inductance increment at elevated temperatures indicated that the steel cable was approaching magnetic saturation. Under such conditions, the enhancement effect of the domain wall motion progressively stopped, reducing the rate of inductance variation. The experimental results exhibited excellent reproducibility, and the three tests demonstrated high consistency, with a relative deviation of up to 2.5%. This robustly validated both the reliability and repeatability of the electromagnetic response-based method for axial load detection in steel cables. These findings provide critical insights for temperature-compensated force sensing applications in structural health monitoring systems.

The experimental results clearly demonstrate that the inductance of the steel strand exhibits distinct yet consistent behaviors under the combined influence of tension and temperature. As shown in Figure 9, under a constant excitation frequency of 10 kHz, the inductance increases strongly and linearly with tension across all tested temperature conditions—rising from approximately 27.25 μH at 20 kN to about 29.25 μH at 100 kN, with an excellent regression fit (R^2^ > 0.99). This linear behavior is attributed to stress-induced alterations in magnetic domain alignment and geometric deformations, such as the reduction in strand diameter under load. The effect of temperature variation on inductance is comparatively minor: a temperature increase of 30 °C results in a change of only about 0.15 μH in the unstressed state, demonstrating high reversibility and negligible hysteresis. Furthermore, the inductance-temperature profiles during both heating and cooling show similar trends with segment-dependent sensitivity, where the greatest variability occurred in the medium-temperature region (e.g., a difference of 0.017 μH at 40 °C), suggesting a possible thermally sensitive regime for the magnetic material. The inductance-tension curves obtained at different temperatures remain parallel, indicating that the influences of tension and temperature are largely decoupled. This behavior offers a robust foundation for thermal compensation in practical tension-monitoring applications. The high repeatability observed across three experimental trials (with a relative deviation of less than 2.5%) further confirms the reliability and consistency of the magnetoelastic inductance-based method for accurate tension estimation under varying thermal conditions.

### 3.4. Curved Surface Fitting Model for Axial Tension Prediction

**Figure 11:**
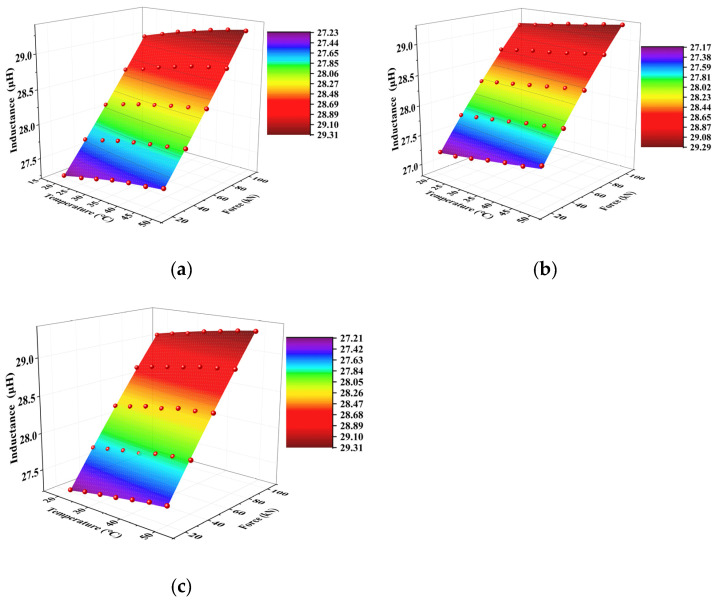
Data fitting model of the three experiments: (**a**) the first test, (**b**) the second test, (**c**) the third test.

The systematic experimental investigations of steel cables under varying temperatures (20–50 °C) and tensile loads (20–100 kN) showed that the inductance parameter demonstrated significant feasibility and reproducibility for axial load characterization. Building upon these findings, the data-driven regression model was established in this study. In the proposed model, surface fitting was performed using temperature and tensile load as independent variables and inductance as a dependent variable. This approach yielded a robust fitting model across the three experimental trials, and detailed results are presented in Figure 11. The proposed model could effectively capture the coupled thermomechanical-electromagnetic behavior, allowing for reliable load estimation through inductance measurements under varying environmental conditions.

**Figure 12:**
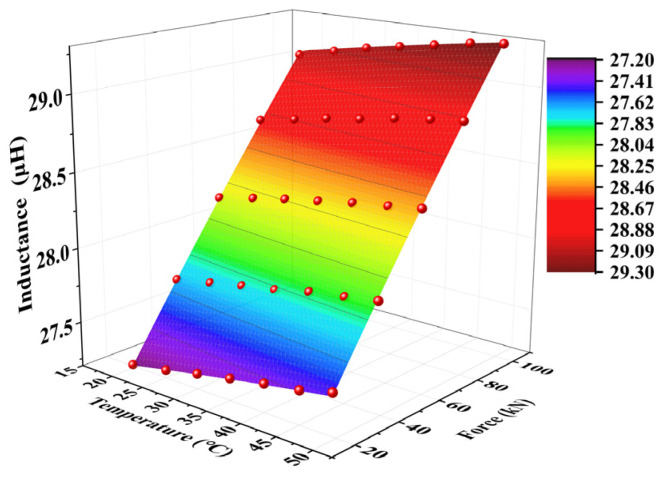
The fitting model of the average values of the three experiments.

The three experimental trials yielded fitting results with high coefficients of determination, where R^2^ > 0.999. This demonstrated the proposed model’s high precision in characterizing inductance variations with respect to both tensile load and temperature. However, to ensure a robust correlation between the inductance and axial load across different temperatures, the experimental data were processed by averaging the temperature, load, and inductance values from the three trials. The averaged parameters were used to develop a final quantitative detection model. The resulting fitted model is shown in Figure 12; this model provided an optimized representation of the temperature-compensated axial load measurement.

The developed surface fitting model demonstrated excellent goodness-of-fit performance, with a coefficient of determination approaching 0.9994. Next, rigorous validation was conducted using other independent measured datasets similar to the experimental results mentioned above that replicated the original environmental conditions. The tensile load values were obtained by inputting the measured temperature and inductance data into the established surface response function. Further, a physics-based solution screening protocol was used to eliminate non-physical multiple solutions. Finally, a systematic comparison between the model-predicted and experimentally applied loads was conducted.

**Figure 13:**
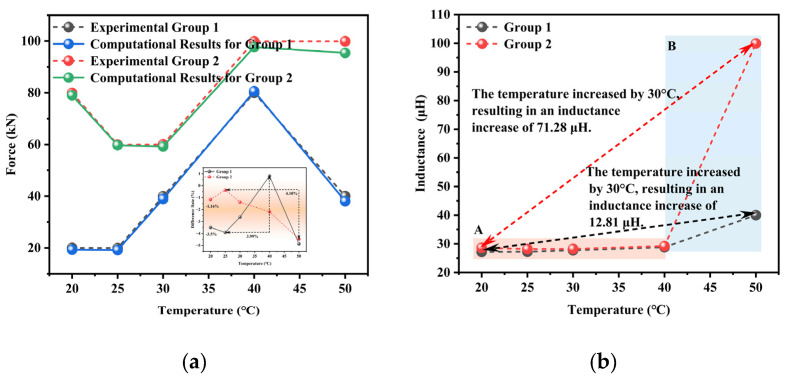
The detection error analysis of the surface fitting model under combined force–temperature conditions: (**a**) force, (**b**) inductance.

The error analysis results of the surface fitting model obtained under combined force–temperature conditions are presented in Figure 13. A comprehensive error analysis was conducted to compare the load values calculated by the quantitative detection model and actual measurement data, and different temperature-dependent error patterns were observed. The experimental data were obtained from laboratory measurements, whereas the theoretical values were calculated using the proposed model. In Experimental Group 1, the error rate exhibited nonlinear variation with temperature increase, namely first decreasing before reaching a peak error of 3.91% and then decreasing again, with a maximum error increment of 3.99% at 60% temperature elevation. The non-monotonic error behavior—initial decrease, peak error of 3.91%, and subsequent decline with temperature rise—stems from competing thermal mechanisms. Initially, reduced viscous damping and enhanced magnetic domain mobility improve response consistency. Around 40% heating duration, non-uniform thermal expansion among components (e.g., cable, sensor core, windings) induces transient misalignments and stress imbalances, elevating error. Beyond this point, the system approaches thermal equilibrium: stress relaxation stabilizes thermal gradients, reducing distortions, while thermal activation of domains and reduced hysteresis enhance signal stability. This behavior highlights the need for effective thermal management and system-wide calibration to ensure reliability across the temperature range. Experimental Group 2 demonstrated different characteristics than Experimental Group 1, showing an initial error increase followed by a reduction, which culminated in a 4.10% error increase at a 100% temperature rise. Fundamentally, both test conditions maintained excellent error control, with all differences falling within the range of −4.87–0.80% and absolute errors remaining below 5%. Temperature-dependent nonlinearity in material permeability increased model deviation at elevated temperatures. In addition, thermal stress gradients induced non-uniform spatial distribution of electromagnetic properties, and measurement system temperature drift introduced additional errors.

Notably, the non-monotonic error–temperature relationship correlated strongly with the material’s nonlinear response characteristics near magnetic saturation. In this study, model validation was performed by inputting multiple independent experimental datasets of Section 3.1 (containing different temperature and inductance values) into the surface fitting model. The resulting load calculations confirmed the proposed model’s accuracy under identical experimental conditions.

### 3.5. BPNN-Based Axial Tension Prediction Model

Considering the characteristic trends and data features observed in the curves, a BPNN architecture was selected for training and prediction of the axial tension of a steel cable. After model development and training, a comprehensive performance evaluation was conducted to quantitatively evaluate the proposed model’s prediction accuracy. The proposed model’s predictive capability was systematically evaluated using two established statistical metrics: mean squared error and coefficient of determination. The mean squared error (MSE) was used to measure the absolute deviation between the predicted and experimental values, and the coefficient of determination (*R*^2^) was employed to evaluate the proportion of variance explained by the model. The MSE value was calculated by:

(5) $$MSE=\frac{1}{n}\sum_{i=1}^{n}{\left(y_{i}-{\hat{y}}_{i}\right)}^{2}$$

where *n* is the total number of samples in a dataset, $y_{i}$ is the actual measured value of the *i*th sample, and ${\hat{y}}_{i}$ is the model-predicted value of the *i*th sample.

The *R*^2^ value was calculated by:

(6) $$R^{2}=1-\frac{\sum_{i=1}^{n}{\left(y_{i}-{\hat{y}}_{i}\right)}^{2}}{\sum_{i=1}^{n}{\left(y_{i}-\bar{y}\right)}^{2}}$$

where $\bar{y}$ is the mean of the measured values.

**Figure 14:**
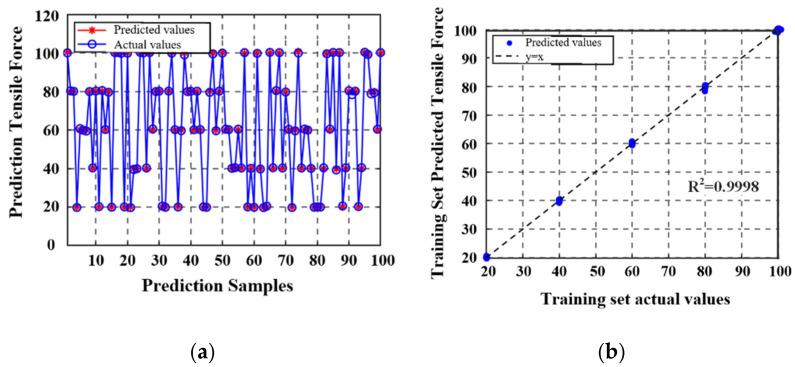
The comparison of predicted and actual values on the training set, *MSE* = 0.152: (**a**) the comparison of predicted and actual values on the training set, (**b**) the predicted values were compared with the actual values in the training set.

**Figure 15:**
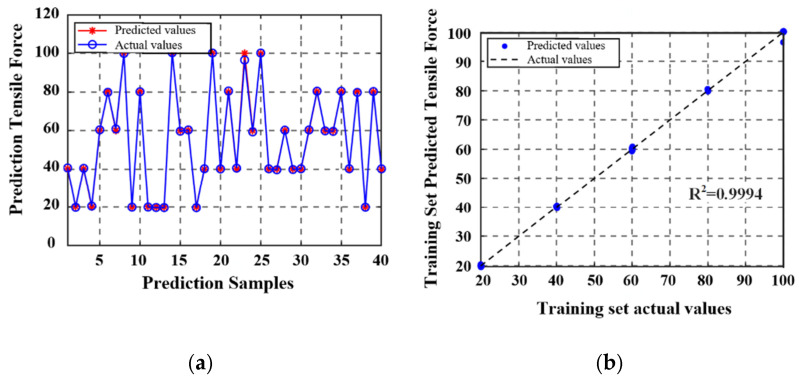
The comparison of predicted and actual values on the test set, *MSE* = 0.4109: (**a**) the comparison of predicted and actual values on the test set, (**b**) the predicted values were compared against the actual values in the test set.

The complete dataset included 140 sets of temperature, inductance, and tensile load measurements and was partitioned into training and testing subsets. Namely, 100 samples were allocated for model training, and the remaining 40 samples were used for independent validation. After model development and evaluation, the prediction performance of the proposed model was visually assessed using comparison plots. The correlation between the predicted and actual values in the training set is presented in Figure 14; the corresponding validation results for the test set are shown in Figure 15.

**Figure 16:**
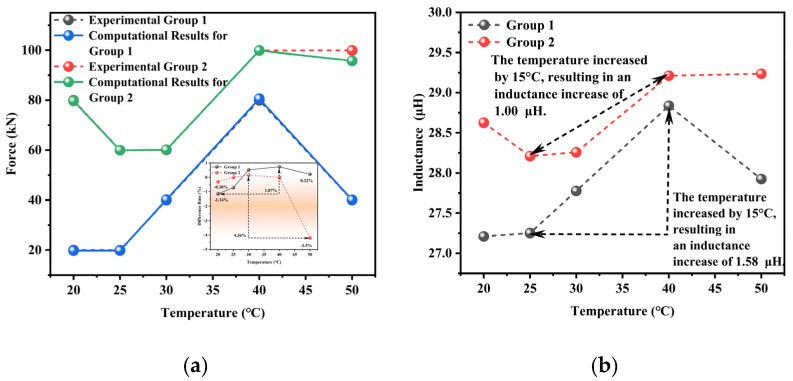
The detection error of the BPNN under force and temperature effects: (**a**) force, (**b**) inductance.

The detection error results of the BPNN-based axial tension prediction model are illustrated in Figure 16, where it can be seen that the first group exhibited a nonlinear variation trend in the error rate with the temperature increase, which was characterized by an initial increase followed by a decrease. The error rate reached a peak value of 1.14% before declining. When the temperature increased from 20 °C to 40 °C, the error rate increased by only 1.87%, which indicated that the system achieved favorable temperature stability in the normal temperature range. In contrast, the second group displayed a markedly different response characteristic; namely, once the temperature exceeded the 40 °C threshold, the error rate exhibited exponential growth, with an increase of 4.36% in the range of 30–50 °C. The tension calculation errors for both operating conditions were within the −3.50–0.73% range, with absolute errors remaining below 4.50%. Compared to the surface fitting model, the BPNN model achieves a reduced error rate, demonstrating higher prediction accuracy.

## 4. Conclusions

To simulate the influence of temperature on the electromagnetic characteristics of drive steel cables used in radiation-resistant robotic systems within nuclear industrial environments, this study conducts experiments using a self-developed force–temperature coupling system to investigate the effects of force and temperature on inductance. In this study, temperature compensation for inductance values is achieved using the surface fitting and BPNN models, improving the accuracy of steel cable force measurement by the magnetoelastic method. However, the calibration of the steel cable force defines the detection accuracy, requiring extensive calibration experiments under conditions that are close to actual working conditions. The experiments are essential for establishing a precise correspondence between temperature, detection parameters, and tensile force values to further enhance measurement accuracy and reliability through algorithmic improvements.

In the axial force measurement of steel cables, inductance signals are prioritized for characterization to ensure higher accuracy. It is shown that minor temperature variations have a limited influence on the inductance of steel cables, and the increasing trends of inductance curves remain consistent across different temperatures. The results show that when the tensile force increases, the inductance demonstrates a regular linear variation. Specifically, when the force increases from 20 kN to 100 kN, the inductance increases from approximately 27.25 μH to 29.25 μH, showing a total increment of approximately 2 μH. Further, the surface fitting and BPNN models are used to predict the axial force of steel cables under varying temperatures. The prediction models’ errors compared to actual force values are all within ±5%, demonstrating the strong compensation capability of the proposed method. This study proposes a novel methodology for analyzing the influence of temperature on the electromagnetic characteristics of drive steel cables used in radiation-resistant robotic systems within nuclear industrial environments. The research contributes to the theoretical foundation for inspecting drive steel cables in these settings. In future studies, the sensor system will be enhanced and subjected to field testing under nuclear industrial environments. Comparative analysis with existing research results will be performed to identify further research directions for this methodology.

## Figures and Tables

**Table 1:** The Parameters of the Materials Used in the Experiments.

Material	Length (mm)	Inside Diameter (mm)	Outer Diameter (mm)	Isobaric Heat Capacity (J/(kg·K)	Thermal Conductivity (W/(m·K))
Foamed silicone rubber hose	400	15.2	30	1900	0.05
Flexible heating tube	400	30	40	–	–
steel cable	500	–	15.2	465	30

**Table 2:** Experimental cable parameters.

Intensity (MPa)	Breaking Force (kN)	Maximum Experimental Tension Value (kN)	Design Tension Value (kN)
1860	260	165	105

**Table 3:** Experimental Parameters Used in the Analysis of Temperature Effects on the Detection Signals under Zero-Load Conditions and Tensile Loading.

Test No.	Temperature (°C)	Excitation Voltage (V)	Measurement Frequency (kHz)	Heating/ Cooling	Detected Signals
1	20–50	30	10	Heating or cooling	Inductance, resistance
2					
3					

## Data Availability

The original contributions presented in this study are included in the article. Further inquiries can be directed to the corresponding author.
